# An efficient and robust approach to Mendelian randomization with measured pleiotropic effects in a high-dimensional setting

**DOI:** 10.1093/biostatistics/kxaa045

**Published:** 2020-11-06

**Authors:** Andrew J Grant, Stephen Burgess

**Affiliations:** MRC Biostatistics Unit, University of Cambridge, Cambridge, UK; MRC Biostatistics Unit, University of Cambridge, Cambridge, UK and Cardiovascular Epidemiology Unit, University of Cambridge, Cambridge, UK

**Keywords:** Causal inference, Instrumental variables, Lasso, Mendelian randomization, Multivariable, Pleiotropy, Summarized data

## Abstract

Valid estimation of a causal effect using instrumental variables requires that all of the instruments are independent of the outcome conditional on the risk factor of interest and any confounders. In Mendelian randomization studies with large numbers of genetic variants used as instruments, it is unlikely that this condition will be met. Any given genetic variant could be associated with a large number of traits, all of which represent potential pathways to the outcome which bypass the risk factor of interest. Such pleiotropy can be accounted for using standard multivariable Mendelian randomization with all possible pleiotropic traits included as covariates. However, the estimator obtained in this way will be inefficient if some of the covariates do not truly sit on pleiotropic pathways to the outcome. We present a method that uses regularization to identify which out of a set of potential covariates need to be accounted for in a Mendelian randomization analysis in order to produce an efficient and robust estimator of a causal effect. The method can be used in the case where individual-level data are not available and the analysis must rely on summary-level data only. It can be used where there are any number of potential pleiotropic covariates up to the number of genetic variants less one. We show the results of simulation studies that demonstrate the performance of the proposed regularization method in realistic settings. We also illustrate the method in an applied example which looks at the causal effect of urate plasma concentration on coronary heart disease.

## 1. Introduction

Instrumental variables can be used to estimate the causal effect of an exposure (also called a risk factor) on an outcome from observational data. A variable is a valid instrument if it is: associated with the risk factor; independent of any confounders of the association between the risk factor and the outcome; and independent of the outcome conditional on the risk factor and confounders. These are the three instrumental variables assumptions (Greenland, 2000).

**Fig. 1.**
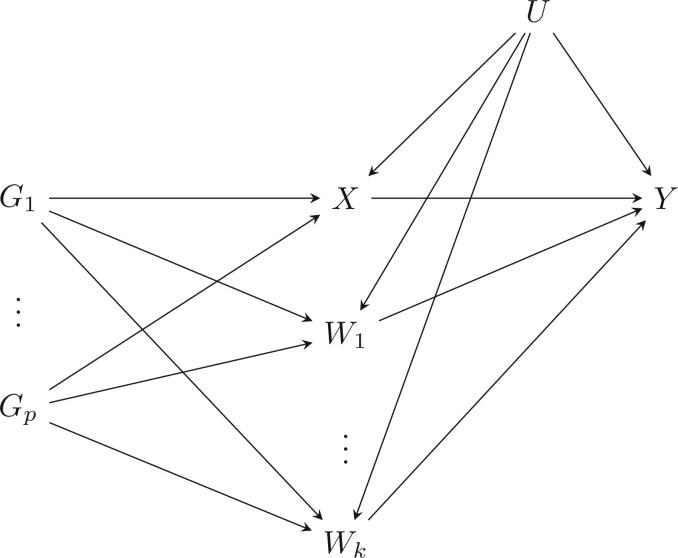
Directed acyclic graph showing the associations between the genetic variants ($G_{1}, \ldots, G_{p}$), the risk factor ($X$), measured covariates which potentially give rise to pleiotropy ($W_{1}, \ldots, W_{k}$), potentially unknown and unmeasured confounders ($U$) and the outcome ($Y$).

In Mendelian randomization studies, genetic variants are used as instrumental variables (Davey Smith and Ebrahim, 2003; Lawlor *and others*, 2008). Although genetic variants have many properties that make them attractive candidates for instruments, one disadvantage is that a single variant typically explains only a small amount of the variation in a risk factor. It is therefore advantageous to combine information from a number of genetic variants. Given the proliferation of genome-wide association studies (GWAS) in recent years, there is data available linking genetic variants across the entire human genome to an enormous number of traits. Standard instrumental variables and meta-analysis techniques allow us to combine the individual estimates given by each of these genetic variants (Thompson and Sharp, 1999; Palmer *and others*, 2012). However, the more genetic variants that are added to an analysis, the more likely that at least one of them will be an invalid instrument. In particular, any given genetic variant could associate with a number of traits other than the risk factor of interest. If any of these traits, which we refer to as covariates, associate with the outcome via pathways that bypass the risk factor, then the third instrumental variables assumption is violated and estimates of the causal effect will be biased. This is known as pleiotropy. This scenario is illustrated via the directed acyclic graph in Figure 1.

There are many methods for estimating a causal effect in the presence of pleiotropy. However, such methods typically require at least some of the genetic variants to be valid instruments. These include median-based estimators (Bowden *and others*, 2016), mode-based estimators (Hartwig *and others*, 2017a; Guo *and others*, 2018) and the contamination mixture method (Burgess *and others*, 2020). The MR-Egger method (Bowden *and others*, 2015) consistently estimates the causal effect without requiring the assumption of no pleiotropy. However, the method relies on a different assumption that pleiotropic effects are independent of the genetic variant-risk factor associations. This assumption is almost as strong as the one it replaces and hypothesis testing based on the MR-Egger method often has low power. Regularization methods proposed by Kang *and others* (2016), Windmeijer *and others* (2019), and Rees *and others* (2019) use $\ell_{1}$ penalization of the least-squares equation to down-weight, and possibly remove, invalid instruments. These methods implicitly assume that at least some of the instruments are valid, and Kang *and others* (2016) show that consistent estimation requires a majority of instruments to be valid.

Jiang *and others* (2019) proposed a constrained optimization approach to construct a weighting scheme for the genetic variants that balances the pleiotropic effects. The weighting scheme can be thought of as weights used to construct an allele score (Burgess and Thompson, 2013; Burgess *and others*, 2016), which can then be used as an instrument in place of the genetic variants themselves. As we shall demonstrate, as long as the number of genetic variants is greater than the number of covariates, the estimator obtained in this way is equivalent to that obtained using standard multivariable Mendelian randomization (Burgess and Thompson, 2015). The interpretation of this is that we can account for covariates which give rise to pleiotropy by including them in a multivariable Mendelian randomization model. However, in practice, it will very often be the case that only a relatively small number of potential covariates need to be included in a multivariable analysis in order to balance pleiotropy. That is, only some of the traits will actually sit on pathways to the outcome which bypasses the risk factor. If this is the case, then the estimator of the causal effect obtained by a multivariable Mendelian randomization analysis with all potential covariates included will be inefficient.

In this article, we propose a method for estimating a causal effect where any number of instruments are invalid due to measured pleiotropy. The method identifies the covariates, among a set of potential covariates, which are on causal pathways from the genetic variants to the outcome, and which therefore should be accounted for in a multivariable Mendelian randomization analysis. Our approach is to fit a multivariable model that applies an $\ell_{1}$ penalty on the coefficients of the genetic variant-covariate associations without applying penalization on the coefficient of the genetic variant-risk factor association. The coefficients of the genetic variant associations with the covariates which have little or no pleiotropic effects will be shrunk towards zero. We thus obtain a more efficient estimator of the causal effect than we would by controlling for all covariates, and a less-biased estimate than we would by ignoring the covariates. There are existing methods that apply regularization to covariates (see, e.g., Caner, 2009; Fan and Liao, 2014). The method of Lin *and others* (2015) is in fact a two-stage procedure that regularizes both the covariates and the instruments. Our situation differs from these, however, in that we do not wish to apply penalization to the coefficient of the risk factor, but only those of the covariates. That is, not all coefficients in the model are penalized, which is a non-standard scenario. The approach is developed for use with summarized data, that is, when the only data available are estimates of the genetic associations with the risk factor, covariates, and outcome, and their standard errors. This is typically the way in which GWAS data are made available. We assess the performance of our approach in simulation studies and demonstrate it with an applied example that looks at the effect of plasma urate concentration on coronary heart disease.

## 2. The model

For individual $i$, let $Y_{i}$ be the outcome, $X_{i}$ be the risk factor, $G_{i}=\begin{bmatrix} G_{i1} & \cdots & G_{ip} \end{bmatrix}'$ be genetic variants and $W_{i}=\begin{bmatrix} W_{i1} & \cdots & W_{ik} \end{bmatrix}'$ be covariates potentially on the causal pathway between each of the genetic variants and the outcome. The model we consider is given by

(2.1) \begin{align*} X_{i} &= G_{i}' \beta_{X} + \gamma_{X} U_{i} + \varepsilon_{Xi} \label{eq:X}\\ \end{align*}

(2.2) \begin{align*} W_{ij} &= G_{i}' \beta_{Wj} + \gamma_{Wj} U_{i} + \varepsilon_{Wij}, \quad j=1,\ldots,k \label{eq:Wj} \\ \end{align*}

(2.3) \begin{align*} Y_{i} &= \theta X_{i} + W_{i}' \delta + \gamma_{Y} U_{i} + \varepsilon_{Yi} \label{eq:Y}, \end{align*}

where $\beta_{X}$ is a $p \times 1$ vector of regression coefficients representing the associations between the $p$ genetic variants and the risk factor and $\beta_{Wj}$ is a $p \times 1$ vector of regression coefficients representing the associations between the $p$ genetic variants and the $j$th covariate. The variable $U_{i}$ represents confounders of the associations between the risk factor, covariates, and outcome. The parameters $\theta$, $\gamma_{X}$, $\gamma_{W1}, \ldots, \gamma_{Wk}$, and $\gamma_{Y}$ are scalars and $\delta = \begin{bmatrix} \delta_{1} & \cdots & \delta_{k} \end{bmatrix}'$ is a $k \times 1$ vector. Note that (2.1)–(2.3) implies that $Y_{i}$ and $G_{i}$ are related by

$$Y_{i} = G_{i}' \beta_{Y} +
\left( \theta \gamma_{X} + \gamma_{W} \delta + \gamma_{Y} \right) U_{i} +
\left( \theta \varepsilon_{Xi} + \varepsilon_{Wi} \delta + \varepsilon_{Yi}\right),$$

where $\beta_{Y} = \theta \beta_{X} + \beta_{W} \delta$, $\beta_{W} = \begin{bmatrix} \beta_{W1} & \cdots & \beta_{Wk} \end{bmatrix}$, $\gamma_{W} = \begin{bmatrix} \gamma_{W1} & \cdots & \gamma_{Wk} \end{bmatrix}$ and $\varepsilon_{Wi} = \begin{bmatrix} \varepsilon_{Wi1} & \cdots & \varepsilon_{Wik} \end{bmatrix}$.

We make the following assumptions.

1. The noise terms, $\varepsilon_{Xi}, \varepsilon_{Wi1}, \ldots, \varepsilon_{Wik}$, and $\varepsilon_{Yi}$ are independent of $U_{i}$ and the genetic variants.
2. The genetic variants are independent of each other (i.e., no linkage disequilibrium) and independent of $U_{i}$.
3.
$p>k$ and the matrix $\begin{bmatrix} \beta_{X} & \beta_{W} \end{bmatrix}$ is of full column rank.

In practice, a set of genetic variants can always be pruned to be in linkage equilibrium by including only one variant per gene region, and thus to satisfy Assumption 2. Assumption 3 ensures that $\theta$ is identifiable (Sanderson *and others*, 2019).

We let $\hat{\beta}_{Xi}$, $\hat{\beta}_{Wij}$, and $\hat{\beta}_{Yi}$ be the estimates of the associations between the $i$th genetic variant and the risk factor, the $j$th covariate and the outcome, respectively. We denote by $\hat{\beta}_{X}$ and $\hat{\beta}_{Y}$ the $p \times 1$ vectors with $i$th elements $\hat{\beta}_{Xi}$ and $\hat{\beta}_{Yi}$, respectively, and $\hat{\beta}_{W}$ the $p \times k$ matrix with $\left( i,j \right)$th element $\hat{\beta}_{Wij}$. While instrumental variable analyses can be performed using individual-level data, often in practice only summarized data in the form of these regression coefficients and their standard errors are available to investigators. To aid applicability of the method, our method is formulated using these summarized data only.

If the three instrumental variables assumptions are met, the causal effect parameter $\theta$ can be consistently estimated using the two-stage least squares method. In the first stage, the risk factor is regressed on the genetic variants. In the second stage, the outcome is regressed on the fitted values from the first stage. The regression estimate from the second stage is the estimate of the causal effect. When only summarized data are available, the same estimator can be obtained by using the inverse-variance weighted method (Burgess *and others*, 2013), which fits the regression model

$$\hat{\beta}_{Yj} = \theta \hat{\beta}_{Xj} + \varepsilon_{j},$$

where $\varepsilon_{j}$ is assumed to be normally distributed with mean zero and variance equal to the variance of $\hat{\beta}_{Yj}$, denoted ${ \mathrm{se} }^{2} \left( \hat{\beta}_{Yj} \right)$. That is, the inverse-variance weighted estimator is

$$\hat{\theta}_{IVW} = \frac{\hat{\beta}_{X}' S \hat{\beta}_{Y}}{\hat{\beta}_{X}' S \hat{\beta}_{X}},$$

where $S$ is the $p \times p$ diagonal matrix with $(j,j)$th element ${ \mathrm{se} }^{-2} \left( \hat{\beta}_{Yj} \right)$. Under the model considered here,

$$\hat{\theta}_{IVW} \rightarrow_{p} \theta + \frac{\beta_{X}' \Sigma_{G} \beta_{W} \delta}{\beta_{X}' \Sigma_{G} \beta_{X}},$$

where $\rightarrow_{p}$ denotes convergence in probability and $\Sigma_{G}$ is the $p \times p$ matrix with $\left( i,j \right)$th element the covariance of the $i$th and $j$th genetic variant. Thus, $\hat{\theta}_{IVW}$ is an inconsistent estimator of the causal effect if $\beta_{W} \delta \neq 0$, that is, if pleiotropy is present.

We can account for the pleiotropy resulting from the measured covariates using the multivariable inverse-variance weighted method (Burgess *and others*, 2015), which fits the weighted multiple linear regression model

(2.4) \begin{equation*} \hat{\beta}_{Yj} = \theta \hat{\beta}_{Xj} + \delta_{1} \hat{\beta}_{Wj1} + \cdots + \delta_{{k}} \hat{\beta}_{Wjk} + \varepsilon_{j}, \label{eq:mvmr} \end{equation*}

where $\varepsilon_{j}$ is normally distributed with mean zero and variance ${ \mathrm{se} }^{2} \left( \hat{\beta}_{Yj} \right)$ (see Section S1 in the Supplementary material available at *Biostatistics* online for further details and how this relates to the covariate balancing approach of Jiang *and others*, 2019). Thus, we obtain an estimator of the causal effect which controls for measured pleiotropy by using a standard multivariable Mendelian randomization approach. However, as noted above, this estimator will be inefficient if any of the covariates do not sit on pathways between the genetic variants and the outcome which bypass the risk factor.

## 3. The regularization method

### 3.1. Estimating the causal effect

Suppose we believe that not all $k$ covariates have pleiotropic effects. That is, that some of the $\delta_{j}$’s are zero. We can induce sparsity in $\delta$ by including an $\ell_{1}$ penalty term in the least squares equation used for estimating the parameters in (2.4). That is, the parameter estimators are given by

(3.5) \begin{equation*} arg\,min_{\theta, \delta} \frac{1}{2}\left( \hat{\beta}_{Y} - \theta \hat{\beta}_{X} - \hat{\beta}_{W} \delta \right)' S \left( \hat{\beta}_{Y} - \theta \hat{\beta}_{X} - \hat{\beta}_{W} \delta \right) + \lambda \sum_{i=1}^{k} \left| \delta_{i} \right|\!, \label{eq:kreg} \end{equation*}

where $\lambda>0$ is a tuning parameter. This is not a standard Lasso problem, since we are not penalizing all the parameters in the model. It is analogous to the some valid, some invalid IV estimator (sisVIVE) of Kang *and others* (2016), which also minimizes a sum of squares function with all but one parameter subject to penalization. In the sisVIVE setup, invalid instruments are identified by applying penalization on direct effects between the instruments and the outcome, but the causal effect is not subject to penalization. Our case is different in that we do not seek to identify valid instruments, but rather to identify pleiotropic covariates using summarized data.

Following a similar procedure to that of the proof of Theorem 3 in Kang *and others* (2016), it is shown in Section S2 of the Supplementary material available at *Biostatistics* online that the estimator of $\theta$ obtained by (3.5), for a given value of $\lambda$, is equivalent to that given by the following two-step procedure.

1. Let
  $$\hat{\delta}_{\lambda} = arg\,min_{\delta} \left( \hat{\beta}_{Y} - \hat{\beta}_{W} \delta \right)' S^{1/2} P_{b^{\bot}} S^{1/2} \left( \hat{\beta}_{Y} - \hat{\beta}_{W} \delta \right) + \lambda \sum_{i=1}^{k} \left| \delta_{i} \right|\!,$$
  where $P_{b^{\bot}} = I_{p} - S^{1/2} \hat{\beta}_{X} \left( \hat{\beta}_{X}' S \hat{\beta}_{X} \right)^{-1} \hat{\beta}_{X}' S^{1/2}$.
2. Let
  $$\hat{\theta}_{\lambda} = \frac{\left( \hat{\beta}_{Y} - \hat{\beta}_{W} \hat{\delta}_{\lambda} \right)' S \hat{\beta}_{X}}{\hat{\beta}_{X}' S \hat{\beta}_{X}}.$$

The first step is now a standard Lasso problem. It induces shrinkage on the elements of $\delta$, but not on $\theta$. Some of the elements of $\delta$ will be shrunk to zero, and the corresponding covariates are effectively removed from the analysis. The second step can be interpreted as estimating $\theta$ by a weighted regression of $\hat{\beta}_{Y} - \hat{\beta}_{W} \hat{\delta}_{\lambda}$ on $\hat{\beta}_{X}$. Note that (3.5) has a unique solution since the columns of the design matrix in the Lasso component of the two-step procedure are continuous variables (Tibshirani, 2013).

An alternative estimator is obtained by dropping the covariates that are assigned a zero coefficient by the above procedure and then performing a standard multivariable analysis including the remaining covariates. That is, the two-step procedure is effectively used as a model selection technique. This is along the lines of, for example, the post-Lasso estimators of Belloni *and others* (2012) and Windmeijer *and others* (2019), and the LARS-OLS hybrid estimator of Efron *and others* (2004). The main argument for using such post-regularization estimators is that they avoid potential bias that may arise from the shrinkage of some of the regression coefficients. The cost is some loss of efficiency.

An important consideration of the method is the choice of tuning parameter, $\lambda$, which controls the level of sparsity. In Section S3 of the Supplementary material available at *Biostatistics* online, we discuss strategies for choosing this from the data using $K$-fold cross-validation.

### 3.2. Two-sample Mendelian randomization

An advantage of using summarized data is the possibility of using a two-sample design for Mendelian randomization. Under this design, the genetic variant-risk factor associations and genetic variant-outcome associations are obtained from separate studies, assumed to be non-overlapping and with similar underlying populations (Hartwig *and others*, 2017b). This allows for many combinations of risk factors and outcomes to be considered, since we do not require each trait to have been included in the same study. It also helps to mitigate against the so-called “winner’s curse” (Taylor *and others*, 2014), which causes effect estimates to tend to be overestimated in single sample designs.

In the multivariable setting, a two-sample approach may in fact involve many samples, with up to one extra sample for each covariate. Again, this is a very flexible design in that it allows for any trait that has been included in published GWAS data to be considered as a potential pleiotropic covariate. It is a valid approach as long as each sample is non-overlapping with the genetic variant-outcome sample and is drawn from a similar underlying population. In practice, these conditions may be somewhat restrictive, particularly in a high-dimensional setting where there are many covariates chosen from a number of GWAS datasets. Some studies are included in the datasets of multiple GWAS consortia, and so there may be overlap with the genetic variant-outcome sample. The extent to which any overlap exists should be checked to ensure it is not substantial. Note that these issues are potential limitations of multivariable Mendelian randomization generally.

### 3.3. Inference

Having estimated the causal effect, it is natural to wish to then perform inference, for example, via producing confidence intervals. The post-regularization method will produce a standard error for the causal effect; however, the uncertainty is likely to be underestimated since it does not take in to account the model selection event. The fundamental problem is that the same data are being used to both select the covariates to be analyzed and to do the analysis itself. A simple and pragmatic approach to get around the problem is to use data splitting, which is a practice that goes back (at least) as far as Cox (1975). The idea is to randomly split a dataset into two. One set is used for model selection, the other for inference. The obvious drawback is a loss of power, since the sample size is effectively halved. In our setting, since we are using summarized data, data splitting is not an option. However, using the same logic, we can propose a three sample study design. Here, an independent set of genetic associations is used to perform the regularization method to identify the covariates that should be accounted for. A standard two-sample multivariable Mendelian randomization analysis is then performed using separate datasets that contain genetic variant associations with the identified covariates, risk factor, and outcome. The independent dataset used for covariate selection should be from a sample that is non-overlapping with those in the analysis datasets and from a similar underlying population.

There is a growing literature on methods for performing inference post-model selection without requiring independent samples. Berk *and others* (2013) (see also Bachoc *and others*, 2019) propose controlling the family-wise error rate across all possible models. In this way, correct coverage of confidence intervals is guaranteed. The same data can be used for both model selection and inference, and furthermore, any selection technique can be used, even post-hoc, non-data driven ones. It is, however, very conservative. Furthermore, it is computationally intensive to compute the critical values (the authors note that it begins to be infeasible with more than $20$ covariates).

Another strain of literature proposes the selective inference approach (Lee *and others*, 2016; Tibshirani *and others*, 2016; Taylor and Tibshirani, 2018), where inference is performed conditional on a particular model being chosen. Lee *and others* (2016) present a method for computing confidence intervals specifically for the case where the model has been chosen using Lasso. They show that the distribution of the parameter estimators conditional on the model selection event is a truncated normal. The confidence intervals can be very wide when the parameter estimate is close to the boundaries of the truncated normal, which will tend to occur when the signal is weak. It could be expected that this is the case in our Mendelian randomization setting when instruments are typically weak and the number of instruments is moderate. Furthermore, the method is derived for fixed $\lambda$, and so is not valid if the tuning parameter is computed using the data under analysis, for example using cross-validation.

Another approach is to use a double estimation procedure (Belloni *and others*, 2014). Under this approach, two model selections are performed using standard Lasso. The first selects covariates in the model that regresses $\hat{\beta}_{X}$ on $\hat{\beta}_{W}$. The second selects covariates in the model that regresses $\hat{\beta}_{Y}$ on $\hat{\beta}_{W}$. The set of covariates used in the final model is the union of the two individual sets. The procedure was developed for the scenario where the covariates are determinants of both the risk factor and the outcome. Although this is not the case in the model described in Section 2, in practice there may be associations between the covariates and the risk factor, in which case this method would account for those. In any case, it should provide more conservative confidence intervals than the two-sample post-regularization approach.

## 4. Simulations

Data on 20 000 individuals were generated from the model given in (2.1), (2.2), and (2.3), with $G_{ij}$ simulated from the $\text{Binomial} \left( 2, \pi \right)$ distribution and $U_{i}, \varepsilon_{Xi}, \varepsilon_{Wij}, \varepsilon_{Yi}$ simulated independently from the $N \left( 0, 1 \right)$ distribution. Two scenarios were considered: $p=10$, with $k=8$ (Scenario 1); and $p=80$, with $k=70$ (Scenario 2). We set $\pi=0.3$, $\gamma_{X} = \gamma_{Y} = 1$ and $\gamma_{W1} = \cdots = \gamma_{Wk} = 1/k$. The elements of $\beta_{X}$ were simulated uniformly on the interval $ \left( 0.15, 0.3 \right) $ (Scenario 1) or $\left( 0.05, 0.12 \right)$ (Scenario 2). The elements of the $\beta_{Wj}$’s were simulated uniformly on the interval $\left( -0.2, 0.4 \right)$ (Scenario 1) or $\left( -0.1, 0.15 \right)$ (Scenario 2). These values give average $R^2$ statistics (i.e., the proportion of the variance in the risk factor explained by the genetic variants) of $10.0\%$ (Scenario 1) and $11.7\%$ (Scenario 2). The number of covariates representing pleiotropic pathways (i.e., the number of $\delta_{j}$’s not equal to zero) was either 1, 2, or 4 in Scenario 1, and either 7, 21, or 35 in Scenario 2. The non-zero $\delta_{j}$’s were simulated uniformly on the interval $\left( -0.2, 0.3 \right)$. Note that all instruments in this setting are potentially invalid and the pleiotropy is unbalanced. The causal effect was either $\theta = 0.2$ or $\theta = 0$.

For each scenario and combination of parameters, two independent datasets were generated. In order to produce the summarized data, the genetic variant-risk factor/outcome associations were estimated using simple linear regression on each genetic variant in turn using the first dataset. The estimates of the genetic variant-outcome associations, and their standard errors, were produced in the same way using the second dataset. For each of $1000$ replications, the causal effect was estimated using the following methods.

1. The inverse-variance weighted method (i.e., ignoring all covariates) (IVW).
2. The two step regularization procedure (Reg).
3. The multivariable inverse-variance weighted method including only the covariates given a non-zero coefficient by the two step regularization procedure (Post-reg).
4. The multivariable inverse-variance weighted method with all covariates included (MV-All).
5. The multivariable inverse-variance weighted method with only truly pleiotropic covariates included (Oracle).

When using the regularization procedure (i.e., in methods 1 and 2), the Lasso component of Step 1 was performed using the glmnet package in R (Friedman *and others*, 2010). The set of $\lambda$ values used for cross-validation was the set generated by that package with the number of values set at 100. The inverse-variance weighted and the multivariable inverse-variance weighted methods using the relevant set of covariates (i.e., as used in methods 3, 4, and 5), were performed using the MendelianRandomization package in R (Yavorska and Burgess, 2017). The mean and standard deviations of the estimates are shown in Table 1. Figure S1(a)–(b) in Section S4 of the Supplementary material available at *Biostatistics* online plots the mean squared error for each scenario and method.

**Table 1.** Mean and standard deviation (SD) of estimates from the various estimation methods. Scenario 1 ($p=10$) has $k=8$ covariates of which either 1, 2, or 4 are truly pleiotropic. Scenario 2 ($p=80$) has $k=70$ covariates of which either 7, 21, or 35 are truly pleiotropic.

		$\theta = 0.2$						$\theta = 0$					
		1 / 7 Covariates		2 / 21 Covariates		4 / 35 Covariates		1 / 7 Covariates		2 / 21 Covariates		4 / 35 Covariates	
$p$	Method	Mean	SD	Mean	SD	Mean	SD	Mean	SD	Mean	SD	Mean	SD
*Sparsity in the covariate effects on the outcome*													
10	IVW	0.219	0.077	0.240	0.103	0.289	0.146	0.024	0.075	0.040	0.105	0.086	0.142
	Reg	0.204	0.060	0.203	0.066	0.217	0.090	0.005	0.053	0.007	0.063	0.012	0.088
	Post-reg	0.201	0.066	0.198	0.073	0.210	0.096	0.004	0.059	0.003	0.070	0.006	0.099
	MV-All	0.198	0.282	0.188	0.239	0.196	0.252	0.007	0.259	0.000	0.209	-0.015	0.314
	Oracle	0.199	0.030	0.198	0.037	0.198	0.058	0.000	0.027	0.001	0.033	0.000	0.048
80	IVW	0.290	0.110	0.478	0.194	0.678	0.243	0.095	0.110	0.279	0.199	0.480	0.238
	Reg	0.200	0.050	0.214	0.080	0.231	0.113	0.014	0.045	0.035	0.079	0.053	0.106
	Post-reg	0.181	0.060	0.184	0.088	0.185	0.121	0.003	0.055	0.013	0.084	0.018	0.117
	MV-All	0.167	0.178	0.169	0.194	0.160	0.223	-0.005	0.157	0.003	0.192	0.001	0.211
	Oracle	0.192	0.032	0.189	0.054	0.180	0.083	0.002	0.029	0.004	0.050	0.006	0.078
*Sparsity in the genetic effects on the covariates*													
10	IVW	0.219	0.077	0.240	0.104	0.288	0.146	0.020	0.076	0.040	0.103	0.089	0.146
	Reg	0.203	0.046	0.204	0.057	0.215	0.089	0.003	0.044	0.005	0.055	0.013	0.086
	Post-reg	0.201	0.047	0.200	0.061	0.207	0.094	0.001	0.044	0.002	0.057	0.005	0.092
	MV-All	0.197	0.176	0.199	0.172	0.204	0.199	0.000	0.164	0.000	0.146	0.002	0.171
	Oracle	0.199	0.032	0.198	0.039	0.198	0.060	0.000	0.028	-0.001	0.035	-0.001	0.053
80	IVW	0.290	0.112	0.478	0.194	0.678	0.243	0.096	0.112	0.284	0.193	0.484	0.243
	Reg	0.212	0.056	0.237	0.087	0.253	0.118	0.020	0.052	0.047	0.082	0.068	0.112
	Post-reg	0.197	0.053	0.208	0.083	0.207	0.116	0.007	0.049	0.022	0.079	0.027	0.112
	MV-All	0.188	0.124	0.194	0.168	0.175	0.202	-0.001	0.116	0.011	0.158	-0.002	0.187
	Oracle	0.191	0.040	0.189	0.063	0.180	0.092	0.001	0.037	0.006	0.059	0.003	0.086

In each case, both the Reg and Post-reg estimators are less biased than IVW and have lower standard deviations. The regularized estimators also have lower standard deviations than the full multivariable estimator, and typically performed at least as well in terms of bias. The mean squared error plots show that the regularized estimators, across all scenarios, sit below the IVW and full multivariable estimators and above the oracle estimator.

The simulations described above represent scenarios where each of the genetic variants are associated with each covariate, but where only some of the covariates have an association with the outcome (i.e., sparsity in the covariate effects on the outcome). In practice, it will often be that all covariates under consideration are associated with the outcome, but only some of them are associated with the genetic instruments (i.e., sparsity in the genetic variant effects on the covariates). The inclusion of covariates in the inverse-variance weighted estimator which associate with the outcome, but not with any of the genetic instruments, will not result in biased estimates, since a pleiotropic pathway via the $j$th covariate exists only when $\delta_{j}$ and at least one element of $\beta_{Wj}$ are non-zero. However, including such covariates may increase the variance of the estimator of $\theta$. Since the regularization approach is trained to minimize mean squared error, the coefficients for these non-pleiotropic covariates will tend to shrink to zero. Thus, we still expect to select only the covariates which sit on pleiotropic pathways.

In order to demonstrate that our method has the desired behavior in the case where there is sparsity in the genetic effects on the covariates, the simulations were repeated where all elements of $\delta$ were non-zero and covariates were removed in the true model by setting columns of $\beta_{W}$ to zero. The mean and standard deviations of the estimates from these simulations are shown in Table 1. The results are in line with the previous ones.

We next consider performing inference using methods discussed in Section 3.3. Using the same set of simulations as above, confidence intervals were computed by performing the multivariable inverse-variance weighted method using sets of covariates that were chosen from: the regularization procedures, in both two and three sample settings; double estimation; and the oracle. These were also compared with confidence intervals computed using IVW (i.e., ignoring covariates) and MV-All (i.e., including all covariates). The results are shown in Section S4 (Tables S1–S2) of the Supplementary material available at *Biostatistics* online, for both the sparsity in the covariate effects on the outcome and sparsity in the genetic effects on the covariates cases. Using the same data to do both covariate selection and inference results in under-coverage, as expected. The three sample approach gives coverage close to the nominal level of 0.95, particularly in Scenario 2 with a larger number of instruments. The double estimation method, while producing better coverage than the two-sample approach, in most cases did not reach the 0.95 level. As expected, the IVW method always had the lowest coverage and the full multivariable method always had the lowest power.

## 5. Investigating the causal effect of urate concentration on coronary heart disease

We consider the study of White *and others* (2016) looking at the effect of plasma urate concentration on coronary heart disease. The study identified 31 genetic variants associated with urate concentration at a genome-wide significance level of $5 \times 10^{-8}$. Summarized associations between these genetic variants and urate concentrations were produced from a combination of published meta-analyses. Eight potential pleiotropic covariates were identified: Fasting glucose, body mass index (BMI), type 2 diabetes, high-density lipoprotein (HDL) cholesterol, low-density lipoprotein (LDL) cholesterol, triglycerides, systolic blood pressure (SBP), and diastolic blood pressure (DBP). These covariates were chosen as risk factors that have been shown observationally to be associated with increased urate concentration and are also known risk factors for coronary heart disease. By examining the associations between the 31 genetic variants and the covariates, White *and others* (2016) concluded that four of them were potential sources of pleiotropy: HDL cholesterol, triglycerides, SBP, and DBP. A Mendelian randomization analysis ignoring covariates suggests that urate concentration has a causal effect on coronary heart disease. However, when including the covariates in the model, the results suggest that there is no causal effect. This is supported by Bowden *and others* (2017), who analyzed the same data using the MR-Egger method.

**Fig. 2.**
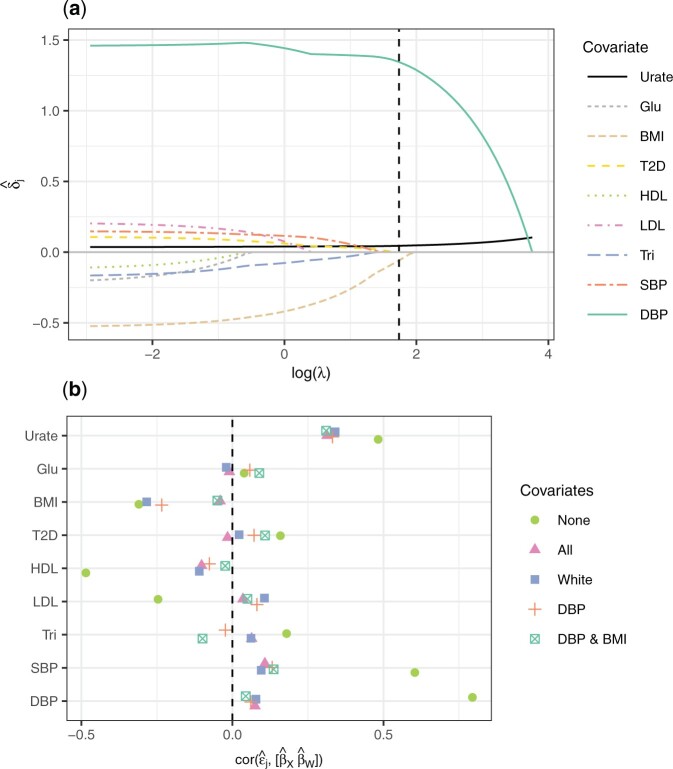
Plots showing the results of applying the regularization method to estimate the effect of urate concentration on coronary heart disease considering eight potential covariates. Plot (a) shows the estimates of regression coefficients for the genetic variant-risk factor association and the genetic variant-covariate associations for different values of $\lambda$. The dashed vertical line indicates the value of $\lambda$ chosen by cross-validation. Plot (b) shows the correlation between the genetic variant-risk factor association and genetic variant-covariate associations, and the residuals obtained after regressing the genetic variant-outcome association on each set of genetic variant-covariate associations.

We re-analyzed the causal effect of urate concentration on CHD using our regularization method. Details of each of the data sources for the genetic variant associations are given in Section S5 of the Supplementary material available at *Biostatistics* online (noting there are some differences in the data sources used here to those used by White *and others*, 2016). Figure 2(a) shows the values of the coefficients for the genetic variant-risk factor and genetic variant-covariate associations produced by the regularization procedure for increasing values of $\lambda$. The value of $\lambda$ used in the final model was chosen by performing 10-fold cross-validation $100$ times and taking the mean minimizer of the mean squared error. This value is indicated in Figure 2(a) by the vertical dashed line. The procedure identified two covariates that should be included in the analysis: DBP and BMI. This suggests that pleiotropy is being caused by these two covariates only. Interestingly, BMI was not identified by White *and others* (2016) as a covariate to be included in the model. HDL cholesterol was the first covariate to be removed by the Lasso, whereas it was one of the four chosen by White *and others* (2016).

We performed multivariable Mendelian randomization analyses using five sets of covariates: no covariates; all covariates; the four covariates identified by White *and others* (2016); DBP only; and DBP and BMI. Table 2 shows the estimates of the log causal odds ratio for each model, as well as their standard errors and 95% confidence intervals (computed using a random-effects model and the normal distribution). In agreement with the previous studies, the results suggest that urate concentration has a causal effect on coronary heart disease when ignoring covariates. When covariates are included, the results suggest that there is no causal effect. The causal effect estimate when only DBP (0.036) or DBP and BMI (0.034) were included are close to the estimates obtained by including all covariates (0.036) or the set of covariates chosen by White *and others* (2016) (0.038).

**Table 2.** Estimates, standard errors (SE), and 95% confidence intervals of the log causal odds ratio for coronary heart disease per one standard deviation increase in plasma urate concentration levels.

Covariates included	Estimate	SE	95% confidence interval
None	0.104	0.040	(0.025 to 0.182)
All	0.036	0.031	($-$0.025 to 0.096)
HDL, Tri, SBP, and DBP	0.038	0.029	($-$0.020 to 0.095)
DBP	0.036	0.027	($-$0.017 to 0.089)
DBP and BMI	0.034	0.027	($-$0.019 to 0.087)

We use a covariate balancing plot which shows the correlation between the genetic variant-risk factor/covariate associations and the residuals obtained after regressing the genetic variant-outcome associations on the genetic variant-covariate associations for each set of covariates considered. If there is no pleiotropy exerted by the covariates, or there is pleiotropy but the model has accounted for it, the correlations with the genetic variant-covariate associations will be close to zero. The plot thus demonstrates two things: the strength of the association between the instruments and the risk factor when controlling for the different sets of covariates (shown by the size of the correlation with urate concentration), and how well each model has balanced the pleiotropic effects (shown by the size of the correlations with the covariates). Figure 2(b) shows that, when all covariates are ignored, the genetic variant-risk factor correlation is the strongest, but there are also strong correlations with all other covariates except for glucose fasting. When all covariates are included, the genetic variant-risk factor strength is somewhat weaker, but all covariates are close to uncorrelated with the residuals. When the four covariates of White *and others* (2016) are included, the covariates are reasonably balanced except for BMI, and a similar pattern is seen when DBP only is included. When DBP and BMI are included, a similar pattern of instrument-exposure correlation and covariate balance is seen as in the case of the full multivariable model.

It should be noted that the analysis here has been performed in a two-sample framework, implying that the confidence interval from the model chosen by the regularization method could be too narrow. This would be the case if further covariates should be included to account for pleiotropy. However, the covariate balancing plot suggests that the inclusion of more covariates is not needed. Furthermore, since we have a finding of no causal effect, widening the confidence interval will only strengthen the evidence behind this finding.

Although not the primary aim of this work, further analysis could be conducted to gain insight into the relationships among the risk factor and covariates chosen by the regularization method (see also Section 6). The relationship between urate concentration, coronary heart disease, blood pressure, and BMI (among other traits) has been studied by Gill *and others* (2020). They showed that urate-lowering treatments reduced blood pressure in randomized trials, suggesting that blood pressure acts as a mediator of the effect of urate concentration on coronary heart disease.

## 6. Extending the causal diagram

**Fig. 3.**
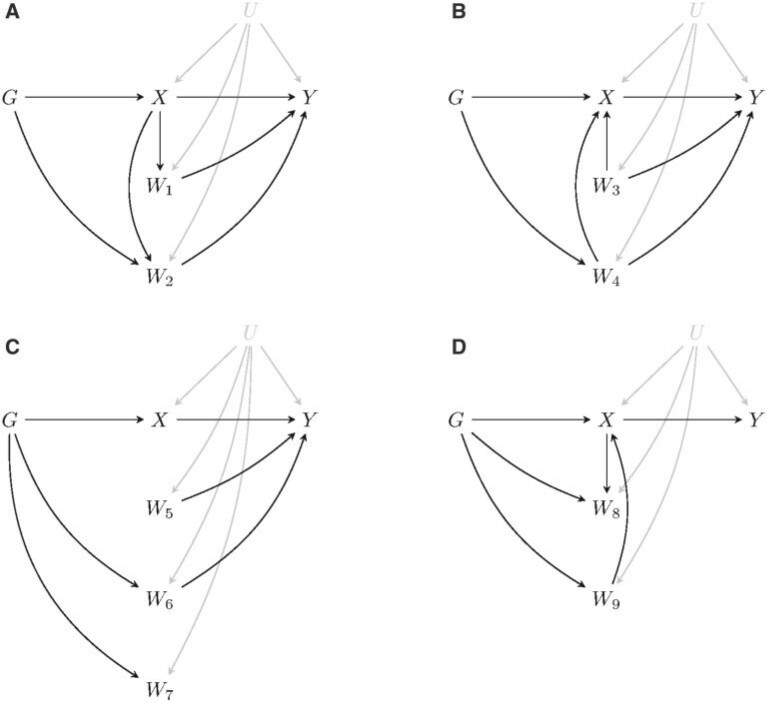
Directed acyclic graphs illustrating the possible causal relationships between the covariates and the genetic variants, risk factor, and outcome. Here, the $G$ and $W_{1}, \ldots, W_{9}$ nodes may represent more than one variable.

In this section, we consider extensions to the model given by (2.1)–(2.3) to allow for causal pathways between the risk factor and covariates. There are nine possible scenarios for a covariate which are illustrated in Figure 3. A covariate may be a mediator of the effect of the risk factor on the outcome (Figure 3(a)); be a confounder of the risk factor-outcome relationship (Figure 3(b)); have no causal pathway to or from the risk factor (Figure 3(c)); or have a causal pathway to or from the risk factor but no association with the outcome (Figure 3(d)).

The regularization method is designed to select covariates which lie on a pathway between the genetic variants and the outcome which bypasses the risk factor. That is, those covariates contained in the $W_{2}$, $W_{4}$, and $W_{6}$ nodes will be selected for inclusion in a multivariable model in order to account for pleiotropy. Mediators which are not independently predicted by the genetic variants (i.e., those in node $W_{1}$) will not be selected. In practice, a mediation analysis (Burgess *and others*, 2017) could be subsequently performed in order to identify which, if any, covariates belong to the $W_{1}$ and $W_{2}$ nodes. If there are no mediators (i.e., no covariates in $W_{1}$ or $W_{2}$), then the direct effect of the risk factor on the outcome will equal the total effect. If there are mediators present, then care needs to be taken over the interpretation of the causal effect estimate.

If covariates are selected in $W_{2}$, the estimand is the direct effect of the risk factor, which includes any effects mediated by non-pleiotropic covariates (i.e., those in $W_{1}$). If the desired target of estimation is the total effect of the risk factor on the outcome, then we must restrict the set of genetic variants to those which are not associated with $W_{2}$ other than via the risk factor, assuming this is possible (Sanderson *and others*, 2019). Burgess *and others* (2017) outline an approach to identify and estimate the direct effect of the risk factor on the outcome free of mediated effects via $W_{1}$. Note that the regularization method does not distinguish between covariates contained in $W_{3}$, $W_{5}$, $W_{7}$, $W_{8}$, and $W_{9}$, since these do not cause pleiotropy or affect the interpretation of the causal estimate.

## 7. Discussion

In this article, we have presented a method for estimating a causal effect of a risk factor on an outcome in a Mendelian randomization setting in the face of pleiotropy. The method does not require any of the genetic variants to be valid instruments and can be performed using summarized data only. By controlling for covariates that have pathways to the outcome which bypass the risk factor, we remove the bias that arises due to these covariates. By not controlling for further covariates unnecessarily, we gain a more precise estimate than we would from a full multivariable model. We also discussed different ways of constructing confidence intervals for the causal effect. Simulations suggest that our proposed three sample approach produces valid confidence intervals and can be used to infer the presence of a causal effect.

The method provides an important tool for sensitivity analysis in a polygenic Mendelian randomization study where it is suspected there is a pleiotropy from some of a given set of covariates. This was demonstrated in the applied example, where we showed that a smaller set of covariates needed to be controlled for than was previously identified.

As with standard multivariable Mendelian randomization, any unmeasured pleiotropy, including from direct effects of the genetic instruments on the outcome, will not be accounted for by the method. However, given the wide availability of genetic association data on deeply phenotyped cohorts (e.g., UK Biobank), it is plausible that in practice any covariates which are believed could potentially result in pleiotropy may be accounted for. As a further sensitivity analysis, a multivariable MR-Egger analysis could be performed using the covariates selected by the regularization procedure (Rees *and others*, 2017). If the causal effect estimate attenuates in the multivariable MR-Egger analysis, this is evidence that there may be further pleiotropic effects that have not been considered. An area for future research is to expand the model given in (3.5) to include intercept terms to account for unmeasured pleiotropy similarly to the method of Kang *and others* (2016) in the univariable setting.

If all potential pleiotropic pathways are accounted for in the given set of covariates, then provided the Lasso step in the regularization procedure selects the truly pleiotropic covariates, the post-regularization estimator will be consistent. In fact, the post-regularization estimator will still be consistent as long as the set of non-zero elements of $\hat{\delta}_{\lambda}$ contains all non-zero elements of the true $\delta$. Meinshausen and Yu (2009) provide conditions under which the Lasso selects, asymptotically, the truly non-zero entries of the coefficient vector along with, possibly, some truly zero entries. For the linear model where the covariates are independent of the error term, this will be the case if the number of covariates remains fixed as the number of observations gets large. Thus, in the two-sample setting, the regularization procedure will tend to select the truly pleiotropic covariates as the number of instruments gets large. Some further discussion related to the consistency of the method is provided in Section S6 of the Supplementary material available at *Biostatistics* online.

The method has been developed to find the most efficient estimator which accounts for measured pleiotropy for a given set of instruments. In practice, this would typically be the set of genetic variants that have been found to associate with the risk factor at the genome-wide significance level, such as in the applied example. By restricting the set of instruments to those with genome-wide significance, we avoid including weak instruments, which could potentially affect estimation. Nonetheless, the simulations reported in Section 4 demonstrate that the method performs well even when the set of instruments contains a large number of reasonably weakly associated genetic variants.

As previously noted, the assumption of independent instruments is not overly restrictive in practice, as a set of genetic variants can always be pruned to be in linkage equilibrium. Even so, the method can be modified to account for correlations between genetic variants. We demonstrate this in Section S7 of the Supplementary material available at *Biostatistics* online.

Although the model we consider allows for correlation among the covariates, we do not consider the potential causal dependencies among these variables. Under linearity assumptions, such causal dependencies will not affect the interpretation of the estimate of $\theta$. They may, however, affect the interpretation of the $\delta_{j}$ estimates. If required, such causal dependencies may be analyzed in subsequent analyses using existing methods (see, e.g., Burgess *and others*, 2015a; Berzuini *and others*, 2020).

The scenarios considered in the article were restricted to the case where the number of genetic variants is greater than the number of covariates under consideration. A topic for future research will be to consider the case where the number of covariates is greater than the number of genetic variants. Some preliminary simulation work suggests that the method may work well in this case. However, further theoretical considerations are required, for example, relating to sparsity assumptions for the $\delta$ vector.

Finally, we note that we are assuming linearity in the relationships between the genetic variants and the risk factor, covariates, and the outcome. Although we have derived the method as though we have a continuous outcome, an advantage of using summarized data is that it allows us to also consider binary outcomes. In this case, the $\hat{\beta}_{Yj}$’s represent estimates of log odds ratios obtained by fitting logistic regression models. Note, however, that, due to the non-collapsibility of the odds ratio, causal effect estimates will tend toward the null (Vansteelandt *and others*, 2011; Bowden *and others*, 2017).

In summary, the method we have developed provides a causal effect estimator in a Mendelian randomization setting where potentially all genetic instruments are invalid due to pleiotropy via a possibly high-dimensional set of covariates. The estimator is robust to measured pleiotropy and more efficient than existing methods.

## 8. Software

R code for performing the proposed method and for generating the simulation results are available at https://github.com/aj-grant/mrcovreg.

## Supplementary Material

kxaa045_Supplementary_Data
