# Supplementary material for: An efficient and robust approach to Mendelian randomization with measured pleiotropic effects in a high-dimensional setting
Source: Biostatistics. 2020 Nov 6;23(2):609–25. doi: 10.1093/biostatistics/kxaa045 (PMC9007434; doi:10.1093/biostatistics/kxaa045)
Supplement: kxaa045_Supplementary_Data [file kxaa045_supplementary_data.pdf]

Biostatistics (2020), **0**, 0, *pp.* 1–17
doi:10.1093/biostatistics/mr˙covreg˙supp

Supplementary material to: An efficient and robust approach to Mendelian randomization with measured pleiotropic effects in a high-dimensional setting

Andrew J. Grant*

MRC Biostatistics Unit, University of Cambridge, Cambridge, UK

andrew.grant@mrc-bsu.cam.ac.uk

Stephen Burgess

MRC Biostatistics Unit, University of Cambridge, Cambridge, UK and Cardiovascular Epidemiology Unit, University of Cambridge, Cambridge, UK

S1. Covariate balancing

Jiang *and others* (2019) proposed weighting the genetic variants in such a way that the pleiotropic effects are balanced out. Such a weighting scheme, however, will tend to reduce the strength of the association between the risk factor and the weighted genetic variants. A constrained optimization approach was therefore proposed, which aims to maximise the covariance between the weighted genetic variants and the risk factor under the constraint that the covariances between the weighted genetic variants and each of the covariates are zero.

We can adapt the constrained optimization approach to the summarized data case as follows. Letting α be a $p \times 1$ vector of weights, $\text{cov}\,(G\alpha, X) = \alpha' \Sigma_G \beta_X$ and $\text{cov}\,(G\alpha, W) = \alpha' \Sigma_G \beta_W$. We

*To whom correspondence should be addressed.

© The Author 2020. Published by Oxford University Press. All rights reserved. For permissions, please e-mail: journals.permissions@oup.com

thus wish to maximise with respect to α the objective function $\alpha'\Sigma_G\hat{\beta}_X$, subject to $\alpha'\Sigma_G\hat{\beta}_W = 0$ and $\alpha'\Sigma_G\alpha = 1$. The second constraint is a normalising condition so that a unique solution is possible. When $p > k$, this can be solved in closed form by

$$\alpha = \tilde{\alpha} = \frac{\xi}{\xi'\Sigma_G\xi}, \tag{S1.1}$$

where

$$\xi = \hat{\beta}_X - \hat{\beta}_W \left(\hat{\beta}_W'\Sigma_G\hat{\beta}_W\right)^{-1}\left(\hat{\beta}_W'\Sigma_G\hat{\beta}_X\right). \tag{S1.2}$$

The causal effect is then estimated by

$$\frac{\tilde{\alpha}'\Sigma_G\hat{\beta}_Y}{\tilde{\alpha}'\Sigma_G\hat{\beta}_X}. \tag{S1.3}$$

In practice, Σ_G is unknown. However, since S is approximately proportional to Σ_G, we can replace Σ_G by S in (S1.1), (S1.2) and (S1.3).

In order to see that this estimator is equivalent to that obtained by the multivariable inverse-variance weighted method, let $\hat{B} = \begin{bmatrix}\hat{\beta}_X & \hat{\beta}_W\end{bmatrix}$. The multivariable inverse-weighted estimator for $\begin{bmatrix}\theta & \delta'\end{bmatrix}'$ is obtained by regressing $S^{1/2}\hat{\beta}_Y$ on $S^{1/2}\hat{B}$, that is,

$$\left(\hat{B}'S\hat{B}\right)^{-1}\left(\hat{B}'S\hat{\beta}_Y\right) = \begin{bmatrix}\hat{\beta}_X'S\hat{\beta}_X & \hat{\beta}_X'S\hat{\beta}_W \\ \hat{\beta}_W'S\hat{\beta}_X & \hat{\beta}_W'S\hat{\beta}_W\end{bmatrix}^{-1}\begin{bmatrix}\hat{\beta}_X'S\hat{\beta}_Y \\ \hat{\beta}_W'S\hat{\beta}_Y\end{bmatrix}.$$

The estimator of θ is the first entry of this vector. By the matrix inversion lemma, the top row of the first term on the right hand side is

$$\begin{bmatrix}\frac{1}{V} & -\frac{1}{V}\left(\hat{\beta}_X'S\hat{\beta}_W\right)\left(\hat{\beta}_W'S\hat{\beta}_W\right)^{-1}\end{bmatrix}$$

where

$$V = \hat{\beta}_X'S\hat{\beta}_X - \left(\hat{\beta}_X'S\hat{\beta}_W\right)\left(\hat{\beta}_W'S\hat{\beta}_W\right)^{-1}\left(\hat{\beta}_W'S\hat{\beta}_X\right).$$

The estimator for θ is thus

$$\frac{\hat{\beta}_X' S\hat{\beta}_Y - \left(\hat{\beta}_X' S\hat{\beta}_W\right)\left(\hat{\beta}_W' S\hat{\beta}_W\right)^{-1}\left(\hat{\beta}_W' S\hat{\beta}_Y\right)}{\hat{\beta}_X' S\hat{\beta}_X - \left(\hat{\beta}_X' S\hat{\beta}_W\right)\left(\hat{\beta}_W' S\hat{\beta}_W\right)^{-1}\left(\hat{\beta}_W' S\hat{\beta}_X\right)}$$
$$= \frac{\left\{\hat{\beta}_X - \hat{\beta}_W\left(\hat{\beta}_W' S\hat{\beta}_W\right)^{-1}\left(\hat{\beta}_W' S\hat{\beta}_X\right)\right\}' S\hat{\beta}_Y}{\left\{\hat{\beta}_X - \hat{\beta}_W\left(\hat{\beta}_W' S\hat{\beta}_W\right)^{-1}\left(\hat{\beta}_W' S\hat{\beta}_X\right)\right\}' S\hat{\beta}_X}$$
$$= \frac{\tilde{\alpha}' S\hat{\beta}_Y}{\tilde{\alpha}' S\hat{\beta}_X}.$$

S2. Derivation of the two step estimation procedure

We wish to find

$$\arg\min_{\theta,\delta} \frac{1}{2}\left(\hat{\beta}_Y - \theta\hat{\beta}_X - \hat{\beta}_W\delta\right)' S\left(\hat{\beta}_Y - \theta\hat{\beta}_X - \hat{\beta}_W\delta\right) + \lambda\sum_{i=1}^{k}|\delta_i|. \tag{S2.4}$$

Following the notation of Kang *and others* (2016), we let $P_M = M\left(M'M\right)^{-1}M'$ for some matrix M with d rows such that $M'M$ is invertible, and $P_{M^\perp} = I_d - P_M$. Note that $P_M P_M = P_{M^\perp}P_{M^\perp} = P_M$, $P_M P_{M^\perp} = 0$, $P_M + P_{M^\perp} = I_d$ and $P_M M = M$. Denoting by $\|\cdot\|_2$ the ℓ_2 norm, (S2.4) can be written as

$$\frac{1}{2}\arg\min_{\theta,\delta}\left\|S^{1/2}\left(\hat{\beta}_Y - \theta\hat{\beta}_X - \hat{\beta}_W\delta\right)\right\|_2^2 + \lambda\sum_{i=1}^{k}|\delta_i|. \tag{S2.5}$$

Let $b = S^{1/2}\hat{\beta}_X$. Then

$$\frac{1}{2}\left\|S^{1/2}\left(\hat{\beta}_Y - \theta\hat{\beta}_X - \hat{\beta}_W\delta\right)\right\|_2^2 + \lambda\sum_{i=1}^{k}|\delta_i|$$
$$= \frac{1}{2}\left\|\left(P_b + P_{b^\perp}\right)S^{1/2}\left(\hat{\beta}_Y - \theta\hat{\beta}_X - \hat{\beta}_W\delta\right)\right\|_2^2 + \lambda\sum_{i=1}^{k}|\delta_i|$$
$$= \frac{1}{2}\left\|P_b S^{1/2}\left(\hat{\beta}_Y - \theta\hat{\beta}_X - \hat{\beta}_W\delta\right)\right\|_2^2 + \frac{1}{2}\left\|P_{b^\perp}S^{1/2}\left(\hat{\beta}_Y - \theta\hat{\beta}_X - \hat{\beta}_W\delta\right)\right\|_2^2 + \lambda\sum_{i=1}^{k}|\delta_i|$$
$$= \frac{1}{2}\left\|P_b S^{1/2}\left(\hat{\beta}_Y - \hat{\beta}_W\delta\right) - \theta S^{1/2}\hat{\beta}_X\right\|_2^2 + \frac{1}{2}\left\|P_{b^\perp}S^{1/2}\hat{\beta}_Y - P_{b^\perp}S^{1/2}\hat{\beta}_W\delta\right\|_2^2 + \lambda\sum_{i=1}^{k}|\delta_i|. \tag{S2.6}$$

The second and third terms of (S2.6) are independent of θ. The first term of (S2.6) can be set to zero for any value of $\delta = \delta^*$ by putting

$$\theta = \frac{\left(\hat{\beta}_Y - \hat{\beta}_W \delta^*\right)' S \hat{\beta}_X}{\hat{\beta}_X' S \hat{\beta}_X}. \tag{S2.7}$$

Thus, (S2.5) can be solved by minimising the second and third terms of (S2.6) with respect to δ, then setting θ according to (S2.7). This is the two step procedure.

S3. The choice of tuning parameter

A common approach to choosing the tuning parameter, λ, from the data is K-fold cross-validation. The set of genetic variants is split into K folds, and the estimation procedure is performed, over a range of λ values, holding out each fold in turn. The λ chosen is that which minimizes the mean, across each fold, of a particular target function. A natural choice for the cross-validation target function is the mean squared error, that is

$$\frac{1}{p}\left(\hat{\beta}_Y - \hat{\beta}_W \hat{\delta}_\lambda - \hat{\theta}_\lambda \hat{\beta}_X\right)' S \left(\hat{\beta}_Y - \hat{\beta}_W \hat{\delta}_\lambda - \hat{\theta}_\lambda \hat{\beta}_X\right). \tag{S3.8}$$

An alternative is to make the choice of λ in Step 1, independent of $\hat{\beta}_X$. That is, the cross-validation target function is

$$\frac{1}{p}\left(\hat{\beta}_Y - \hat{\beta}_W \hat{\delta}_\lambda\right)' S^{1/2} P_{b^\perp} S^{1/2} \left(\hat{\beta}_Y - \hat{\beta}_W \hat{\delta}_\lambda\right). \tag{S3.9}$$

The use of (S3.8) as target function will give the smallest test mean squared error and would be expected to give the more precise estimation. The use of (S3.9) will tend to select more covariates, since any covariate-outcome effects which are mediated through the risk factor will not be discounted. It will tend to therefore be more conservative in the sense that the standard deviation of the estimates will be larger. Unless otherwise specified, the results reported in this paper use cross-validation with $K = 10$ and (S3.8) as target function.

It is common practice to apply the one standard error rule in cross-validation procedures, where the chosen value is the smallest that is no more than one standard deviation above the

λ which minimizes the target function (Hastie *and others*, 2009). Use of this rule will tend to induce more sparsity, and is used, for example, by Kang *and others* (2016). In our case, however, removing a covariate from the analysis that is causing pleiotropy will lead to bias, whereas leaving in a covariate that is not needed will just make the estimator less efficient. Thus, it is preferable to lean on the side of under penalization. This is in contrast to, for example the application in Kang *and others* (2016), and other regularization methods mentioned previously, which induce sparsity on the number of instruments. Leaving in an invalid instrument will cause bias, whereas removing a valid instrument will just lower the power of the method in detecting a causal effect. In Section S4.3, we re-analyse the simulation results reported in Section 4 using the one standard error rule. The performance is generally similar, although the results using the one standard error rule tend to be more biased when there are a larger number of pleiotropic covariates.

S4. Further simulation results

S4.1 *Mean squared error plots*

Figure S1 plots the mean squared error for each scenario and method for the cases presented in Table 1.

S4.2 *Inference*

In this section we show the results of performing inference using methods discussed in Section 3.3. Using the same set of simulations as those presented in Table 1, confidence intervals were computed by performing the multivariable inverse-variance weighted method using sets of covariates which were chosen as follows.

1. All covariates ignored (IVW).
2. The two step regularization procedure using the mean squared error, given by (S3.8), in cross-validation (2 sample(a)).

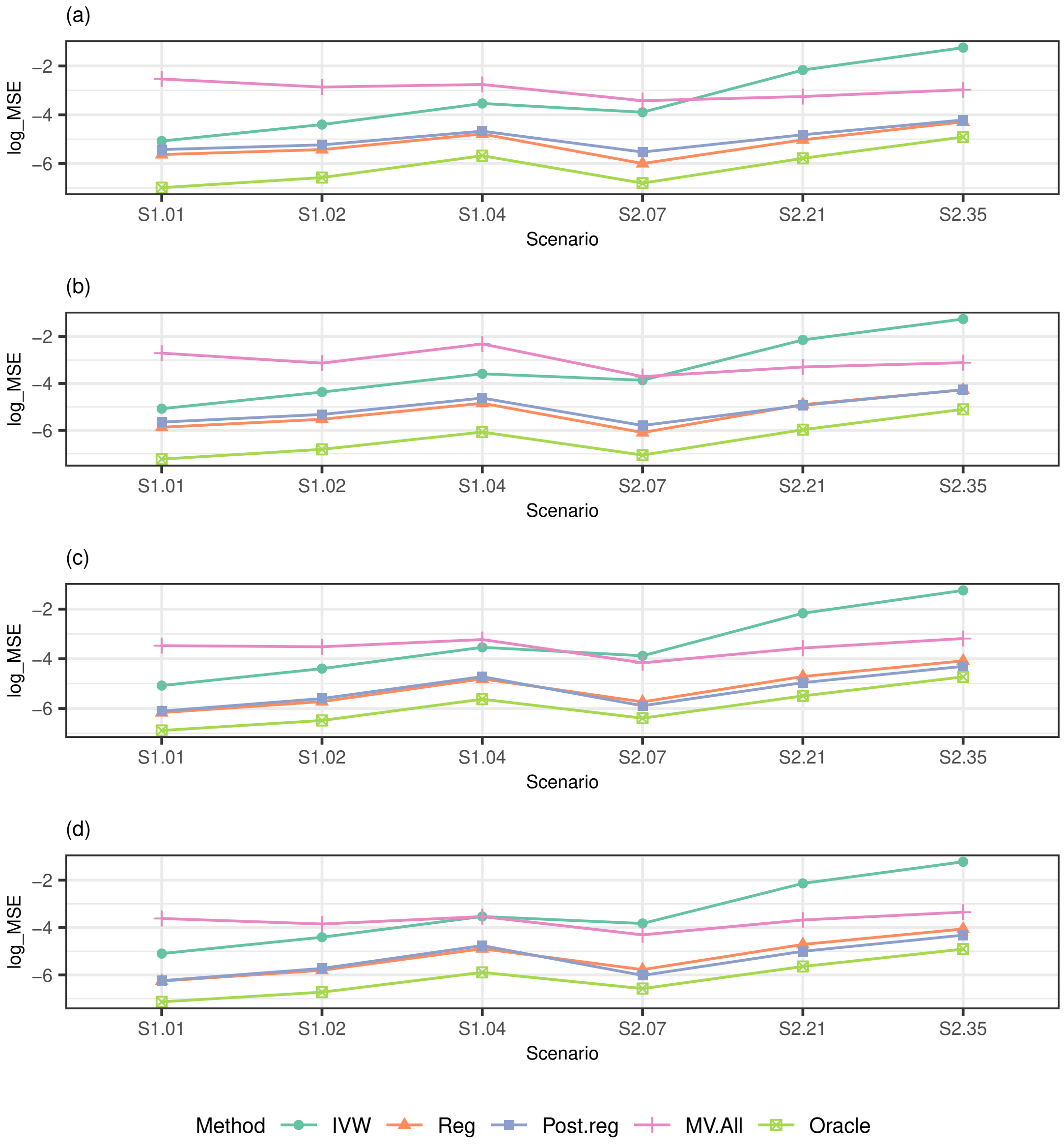

Fig. S1. Logarithm of the mean squared errors for each scenario (S1–S2) and number of truly pleiotropic covariates (01–35). Plots (a) and (b), where $\theta = 0.2$ and $\theta = 0$, respectively, show the results from simulations where there is sparsity in the covariate effects on the outcome. Plots (c) and (d), where $\theta = 0.2$ and $\theta = 0$, respectively, show the results from simulations where there is sparsity in the genetic variant effects on the covariates.

3. The two step regularization procedure where cross-validation was performed independent of the genetic variant-risk factor associations, that is, using (S3.9) as target function (2 sample(b)).
4. The two step regularization procedure using an independent sample and the mean squared error, given by (S3.8), in cross-validation (3 sample(a)).
5. The two step regularization procedure using an independent sample and where cross-validation was performed independent of the genetic variant-risk factor associations, that is, using (S3.9) as target function (3 sample(b)).
6. The double estimation procedure (Double est.).
7. All covariates included (MV-All).
8. Only truly pleiotropic covariates included (Oracle).

In each case, the model was fitted using the MendelianRandomization package in R with random effects (that is, allowing over-dispersion, see Thompson and Sharp, 1999 and Burgess and Thompson, 2017) and 95% confidence intervals derived using the normal distribution. The means of the standard errors, coverage (that is, the proportion of confidence intervals containing the true causal effect) and power (that is, the proportion of confidence intervals not containing zero) are shown in Table S1 (for $\theta = 0.2$) and Table S2 (for $\theta = 0$). Note that in the $\theta = 0$ case, power in fact refers to the Type I error rate.

See Section 4 for discussion of the results. One further point to note is that the use of (S3.9) in cross-validation tends to give coverage closer to 0.95 than the use of (S3.8), although not uniformly. It also gives wider confidence intervals, suggesting that it is more conservative in covariate selection (that is, tends to give lower levels of sparsity).

Table S1. Mean, standard deviation (SD), mean standard errors (SE), coverage (Cov) and power (Pow) of estimates from the various methods with $\theta = 0.2$. Scenario 1 ($p = 10$) has $k = 8$ covariates of which either 1, 2 or 4 are truly pleiotropic. Scenario 2 ($p = 80$) has $k = 70$ covariates of which either 7, 21, or 35 are truly pleiotropic.

		1 / 7 Covariates					2 / 21 Covariates					4 / 35 Covariates				
p	Method	Mean	SD	SE	Cov	Pow	Mean	SD	SE	Cov	Pow	Mean	SD	SE	Cov	Pow
		Sparsity in the covariate effects on the outcome														
10	IVW	0.219	0.077	0.040	0.729	0.958	0.240	0.103	0.054	0.667	0.920	0.289	0.146	0.074	0.595	0.852
	2 sample (a)	0.201	0.066	0.050	0.894	0.922	0.198	0.073	0.056	0.888	0.865	0.210	0.096	0.073	0.859	0.788
	2 sample (b)	0.201	0.146	0.081	0.900	0.794	0.192	0.143	0.082	0.900	0.757	0.209	0.132	0.090	0.883	0.714
	3 sample (a)	0.197	0.074	0.059	0.937	0.891	0.200	0.080	0.065	0.922	0.839	0.208	0.118	0.086	0.914	0.745
	3 sample (b)	0.196	0.213	0.097	0.951	0.763	0.194	0.114	0.094	0.939	0.708	0.205	0.135	0.102	0.928	0.666
	Double est.	0.202	0.100	0.072	0.926	0.804	0.199	0.132	0.094	0.909	0.736	0.203	0.136	0.104	0.892	0.684
	MV-All	0.198	0.282	0.190	0.961	0.425	0.188	0.239	0.196	0.963	0.386	0.196	0.252	0.190	0.957	0.418
	Oracle	0.199	0.030	0.032	0.947	0.999	0.198	0.037	0.039	0.956	0.982	0.198	0.058	0.056	0.953	0.903
80	IVW	0.290	0.110	0.043	0.392	0.970	0.478	0.194	0.069	0.216	0.975	0.678	0.243	0.089	0.081	0.988
	2 sample (a)	0.181	0.060	0.044	0.846	0.923	0.184	0.088	0.069	0.879	0.719	0.185	0.121	0.091	0.848	0.539
	2 sample (b)	0.180	0.062	0.045	0.836	0.908	0.185	0.091	0.070	0.867	0.717	0.185	0.122	0.093	0.853	0.529
	3 sample (a)	0.186	0.051	0.049	0.947	0.936	0.186	0.083	0.084	0.951	0.618	0.186	0.120	0.120	0.941	0.370
	3 sample (b)	0.187	0.051	0.050	0.943	0.938	0.187	0.084	0.084	0.960	0.623	0.186	0.122	0.122	0.931	0.357
	Double est.	0.185	0.058	0.048	0.871	0.918	0.185	0.084	0.073	0.911	0.720	0.191	0.114	0.096	0.895	0.522
	MV-All	0.167	0.178	0.181	0.947	0.153	0.169	0.194	0.200	0.952	0.145	0.160	0.223	0.223	0.943	0.131
	Oracle	0.192	0.032	0.033	0.950	1.000	0.189	0.054	0.054	0.942	0.941	0.180	0.083	0.080	0.939	0.615
		Sparsity in the genetic effects on the covariates														
10	IVW	0.219	0.077	0.041	0.749	0.957	0.240	0.104	0.055	0.679	0.915	0.288	0.146	0.075	0.598	0.851
	2 sample (a)	0.201	0.047	0.039	0.908	0.971	0.200	0.061	0.048	0.883	0.929	0.207	0.094	0.064	0.858	0.835
	2 sample (b)	0.200	0.131	0.057	0.912	0.901	0.200	0.104	0.063	0.902	0.865	0.200	0.122	0.079	0.876	0.737
	3 sample (a)	0.199	0.053	0.048	0.948	0.938	0.198	0.087	0.063	0.936	0.870	0.198	0.102	0.083	0.906	0.748
	3 sample (b)	0.199	0.133	0.071	0.951	0.847	0.196	0.096	0.078	0.954	0.789	0.197	0.146	0.098	0.928	0.679
	Double est	0.201	0.065	0.053	0.925	0.914	0.198	0.114	0.069	0.915	0.836	0.206	0.142	0.092	0.887	0.729
	MV-All	0.197	0.176	0.127	0.956	0.619	0.199	0.172	0.141	0.952	0.565	0.204	0.199	0.154	0.945	0.463
	Oracle	0.199	0.032	0.034	0.951	0.999	0.198	0.039	0.041	0.958	0.977	0.198	0.060	0.058	0.951	0.884
80	IVW	0.290	0.112	0.046	0.432	0.966	0.478	0.194	0.071	0.226	0.975	0.678	0.243	0.089	0.084	0.988
	2 sample (a)	0.197	0.053	0.041	0.872	0.987	0.208	0.083	0.064	0.876	0.837	0.207	0.116	0.087	0.859	0.625
	2 sample (b)	0.198	0.052	0.041	0.869	0.992	0.207	0.085	0.066	0.882	0.815	0.208	0.118	0.088	0.856	0.621
	3 sample (a)	0.193	0.054	0.053	0.948	0.926	0.193	0.088	0.087	0.943	0.627	0.187	0.125	0.122	0.935	0.379
	3 sample (b)	0.193	0.055	0.054	0.942	0.916	0.195	0.089	0.088	0.944	0.614	0.191	0.128	0.123	0.933	0.376
	Double est	0.196	0.053	0.042	0.895	0.978	0.202	0.081	0.067	0.896	0.802	0.205	0.112	0.091	0.887	0.602
	MV-All	0.188	0.124	0.126	0.955	0.362	0.194	0.168	0.168	0.945	0.233	0.175	0.202	0.204	0.953	0.165
	Oracle	0.191	0.040	0.041	0.960	0.996	0.189	0.063	0.063	0.944	0.849	0.180	0.092	0.087	0.929	0.559

Table S2. Mean, standard deviation (SD), mean standard errors (SE), coverage (Cov) and Type I error rate (T I) of estimates from the various methods with $\theta = 0$. Scenario 1 ($p = 10$) has $k = 8$ covariates of which either 1, 2 or 4 are truly pleiotropic. Scenario 2 ($p = 80$) has $k = 70$ covariates of which either 7, 21, or 35 are truly pleiotropic.

		1 / 7 Covariates					2 / 21 Covariates					4 / 35 Covariates				
p	Method	Mean	SD	SE	Cov	T I	Mean	SD	SE	Cov	T I	Mean	SD	SE	Cov	T I
							Sparsity in the covariate effects on the outcome									
10	IVW	0.024	0.075	0.040	0.707	0.293	0.040	0.105	0.053	0.647	0.353	0.086	0.142	0.073	0.587	0.413
	2 sample (a)	0.004	0.059	0.044	0.905	0.095	0.003	0.070	0.053	0.901	0.099	0.006	0.099	0.067	0.863	0.137
	2 sample (b)	0.000	0.171	0.072	0.924	0.076	0.000	0.125	0.075	0.906	0.094	0.001	0.138	0.081	0.874	0.126
	3 sample (a)	-0.003	0.100	0.051	0.936	0.064	0.002	0.065	0.060	0.934	0.066	0.000	0.089	0.074	0.904	0.096
	3 sample (b)	-0.002	0.148	0.086	0.950	0.050	0.007	0.105	0.079	0.942	0.058	0.001	0.110	0.091	0.934	0.066
	Double est.	0.003	0.126	0.072	0.935	0.065	0.003	0.109	0.081	0.929	0.071	-0.007	0.277	0.104	0.916	0.084
	MV-All	0.007	0.259	0.173	0.964	0.036	0.000	0.209	0.163	0.956	0.044	-0.015	0.314	0.170	0.955	0.045
	Oracle	0.000	0.027	0.028	0.964	0.036	0.001	0.033	0.035	0.954	0.046	0.000	0.048	0.050	0.965	0.035
80	IVW	0.095	0.110	0.042	0.376	0.624	0.279	0.199	0.067	0.209	0.791	0.480	0.238	0.087	0.086	0.914
	2 sample (a)	0.003	0.055	0.039	0.845	0.155	0.013	0.084	0.065	0.867	0.133	0.018	0.117	0.087	0.859	0.141
	2 sample (b)	0.002	0.055	0.040	0.846	0.154	0.013	0.086	0.066	0.871	0.129	0.018	0.122	0.089	0.858	0.142
	3 sample (a)	0.005	0.046	0.044	0.931	0.069	0.010	0.083	0.077	0.939	0.061	0.016	0.123	0.112	0.914	0.086
	3 sample (b)	0.005	0.048	0.045	0.934	0.066	0.013	0.083	0.078	0.942	0.058	0.018	0.119	0.113	0.919	0.081
	Double est.	0.006	0.054	0.043	0.865	0.135	0.020	0.080	0.069	0.886	0.114	0.028	0.109	0.092	0.884	0.116
	MV-All	-0.005	0.157	0.161	0.957	0.043	0.003	0.192	0.184	0.946	0.054	0.001	0.211	0.206	0.947	0.053
	Oracle	0.002	0.029	0.030	0.947	0.053	0.004	0.050	0.049	0.943	0.057	0.006	0.078	0.073	0.928	0.072
							Sparsity in the genetic effects on the covariates									
10	IVW	0.020	0.076	0.039	0.735	0.265	0.040	0.103	0.054	0.673	0.327	0.089	0.146	0.074	0.589	0.411
	2 sample (a)	0.001	0.044	0.037	0.929	0.071	0.002	0.057	0.045	0.897	0.103	0.005	0.092	0.060	0.868	0.132
	2 sample (b)	0.000	0.108	0.051	0.926	0.074	0.005	0.090	0.058	0.912	0.088	-0.001	0.111	0.072	0.893	0.107
	3 sample (a)	0.001	0.048	0.043	0.953	0.047	-0.001	0.071	0.057	0.928	0.072	-0.001	0.095	0.075	0.906	0.094
	3 sample (b)	0.001	0.108	0.064	0.960	0.040	-0.002	0.081	0.071	0.951	0.049	-0.002	0.130	0.088	0.930	0.070
	Double est	0.003	0.057	0.048	0.935	0.065	0.001	0.091	0.063	0.926	0.074	0.006	0.104	0.086	0.902	0.098
	MV-All	0.000	0.164	0.114	0.958	0.042	0.000	0.146	0.128	0.958	0.042	0.002	0.171	0.138	0.954	0.046
	Oracle	0.000	0.028	0.030	0.956	0.044	-0.001	0.035	0.037	0.960	0.040	-0.001	0.053	0.052	0.957	0.043
80	IVW	0.096	0.112	0.045	0.397	0.603	0.284	0.193	0.069	0.213	0.787	0.484	0.243	0.088	0.074	0.926
	2 sample (a)	0.007	0.049	0.039	0.869	0.131	0.022	0.079	0.061	0.860	0.140	0.027	0.112	0.083	0.839	0.161
	2 sample (b)	0.007	0.049	0.039	0.870	0.130	0.020	0.080	0.062	0.872	0.128	0.026	0.112	0.084	0.849	0.151
	3 sample (a)	0.003	0.050	0.049	0.937	0.063	0.009	0.083	0.081	0.944	0.056	0.009	0.117	0.114	0.939	0.061
	3 sample (b)	0.003	0.051	0.050	0.939	0.061	0.011	0.084	0.082	0.944	0.056	0.012	0.120	0.115	0.931	0.069
	Double est	0.008	0.049	0.040	0.892	0.108	0.022	0.078	0.064	0.883	0.117	0.033	0.106	0.086	0.868	0.132
	MV-All	-0.001	0.116	0.117	0.956	0.044	0.011	0.158	0.157	0.942	0.058	-0.002	0.187	0.191	0.945	0.055
	Oracle	0.001	0.037	0.038	0.959	0.041	0.006	0.059	0.059	0.935	0.065	0.003	0.086	0.081	0.934	0.066

S4.3 *Use of the one standard error rule*

Table S3 shows the results of the regularization and post-regularization procedures applied to the same simulations as shown in Table 1 but with the one standard error rule applied. Figure S2 plots the mean squared errors for the procedures against those for the results without using the one standard error rule. The use of the one standard error rule results in higher mean squared error when using the regularization procedure. When using the post-regularization procedure, the one standard error rule typically has lower mean squared error with a smaller number of instruments (that is, in scenario 1), and a higher mean squared error with a larger number of instruments (that is, in scenario 2).

S4.4 *Increased correlation among the covariates*

Table S4 shows the results for simulations run under the same parameters as those in Table 1 (when $\theta = 0.2$) but with $\gamma_{Wj} = 0.5$ and $\varepsilon_{Wij} \sim N\left(0, 0.75 + 1/k^2\right)$, $i = 1, \ldots, p$, $j = 1, \ldots, k$. This makes the correlation between each covariate approximately 0.25. The variance of the ε_{Wij} was reduced accordingly so that the signal to noise ratio for each covariate was unchanged.

S4.5 *Simulations with $p = 200$ instruments*

Table S5 shows the results for simulations run under the same parameters as those in Table 1 (when $\theta = 0.2$) but with $p = 200$ and γ_{Wj}, $j = 1, \ldots, k$, set to either $1/k$ or 0.5 (with the variance of the ε_{Wij} adjusted as in Section S4.4).

S5. Data sources for the applied analysis

The associations between the genetic variants and urate concentration were taken from White *and others* (2016). Note that, although the singificance level for inclusion of a genetic variant was

Table S3. Mean and standard deviation (SD) of estimates from the regularization and post-regularization methods, using the one standard error rule. Scenario 1 ($p = 10$) has $k = 8$ covariates of which either 1, 2 or 4 are truly pleiotropic. Scenario 2 ($p = 80$) has $k = 70$ covariates of which either 7, 21, or 35 are truly pleiotropic.

		$\theta = 0.2$						$\theta = 0$					
		1 / 7 Covariates		2 / 21 Covariates		4 / 35 Covariates		1 / 7 Covariates		2 / 21 Covariates		4 / 35 Covariates	
p	Method	Mean	SD	Mean	SD	Mean	SD	Mean	SD	Mean	SD	Mean	SD
Sparsity in the covariate effects on the outcome													
10	Reg (1se)	0.206	0.056	0.212	0.063	0.232	0.095	0.008	0.048	0.012	0.065	0.023	0.093
	Post-reg (1se)	0.201	0.057	0.203	0.064	0.217	0.096	0.003	0.049	0.004	0.065	0.010	0.093
80	Reg (1se)	0.217	0.057	0.245	0.092	0.284	0.130	0.025	0.052	0.059	0.091	0.092	0.121
	Post-reg (1se)	0.187	0.050	0.188	0.083	0.204	0.122	0.003	0.044	0.015	0.077	0.026	0.112
Sparsity in the genetic effects on the covariates													
10	Reg (1se)	0.206	0.051	0.212	0.064	0.231	0.097	0.007	0.047	0.010	0.060	0.031	0.091
	Post-reg (1se)	0.202	0.047	0.203	0.061	0.215	0.097	0.003	0.043	0.001	0.056	0.016	0.089
80	Reg (1se)	0.228	0.069	0.268	0.104	0.308	0.141	0.034	0.065	0.075	0.098	0.117	0.135
	Post-reg (1se)	0.196	0.054	0.207	0.086	0.221	0.123	0.005	0.049	0.022	0.081	0.039	0.116

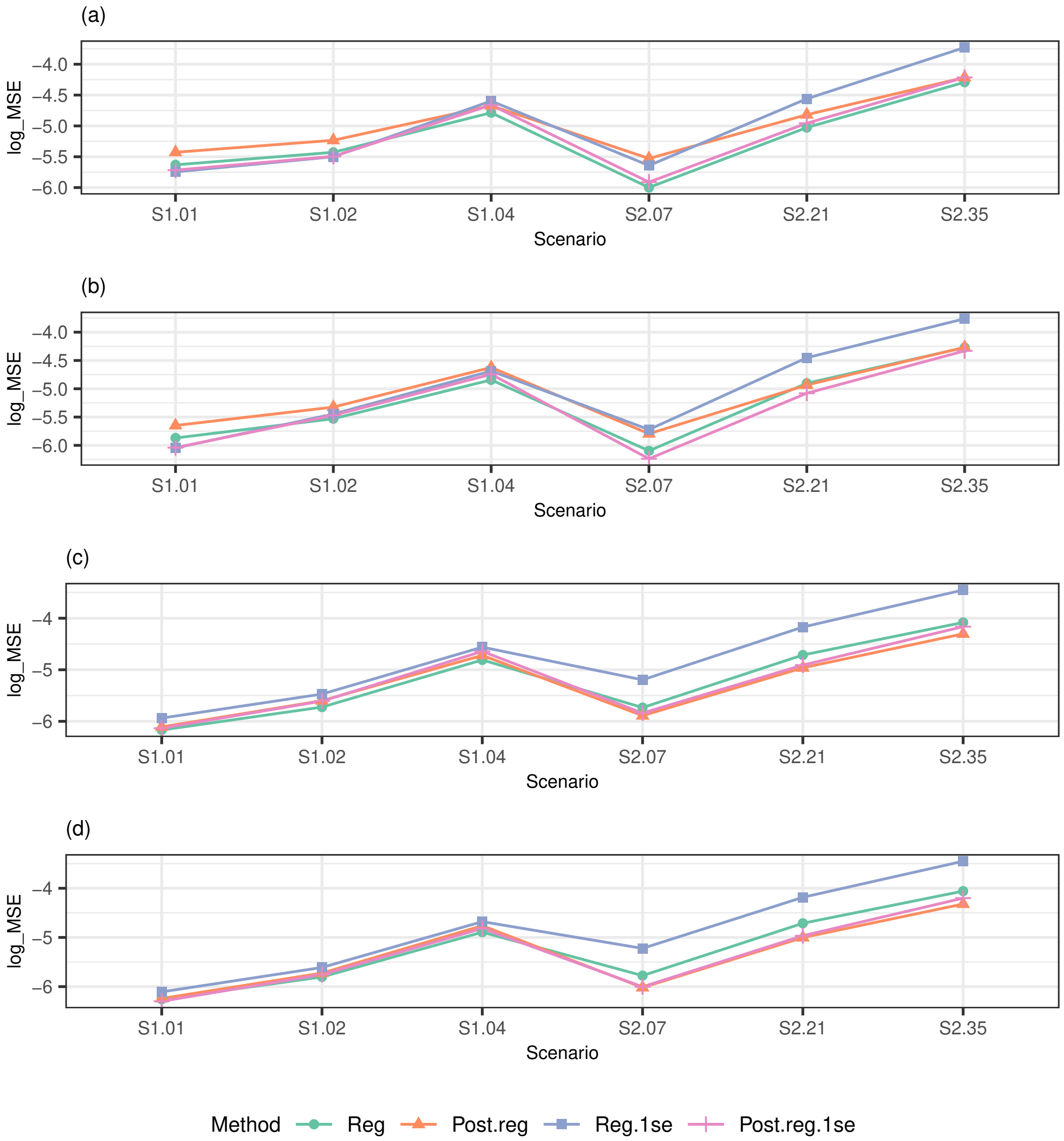

Fig. S2. Logarithm of the mean squared errors for each scenario (S1–S2) and number of truly pleiotropic covariates (01–35), comparing the regularization and post-regularization methods with and without the one standard error rule applied. Plots (a) and (b), where $\theta = 0.2$ and $\theta = 0$, respectively, show the results from simulations where there is sparsity in the covariate effects on the outcome. Plots (c) and (d), where $\theta = 0.2$ and $\theta = 0$, respectively, show the results from simulations where there is sparsity in the genetic variant effects on the covariates.

Table S4. Mean and standard deviation (SD) of estimates from the various methods when there is sparsity in the covariate effects on the outcome, $\theta = 0.2$ and $\gamma_{Wj} = 0.5$, $j = 1, \ldots, k$. Scenario 1 ($p = 10$) has $k = 8$ covariates of which either 1, 2 or 4 are truly pleiotropic. Scenario 2 ($p = 80$) has $k = 70$ covariates of which either 7, 21, or 35 are truly pleiotropic.

		$\theta = 0.2$					
		1 / 7 Covariates		2 / 21 Covariates		4 / 35 Covariates	
p	Method	Mean	SD	Mean	SD	Mean	
10	IVW	0.217	0.077	0.240	0.104	0.290	0.140
	Reg	0.201	0.055	0.211	0.071	0.214	0.089
	Post-reg	0.199	0.059	0.209	0.101	0.203	0.106
	MV-All	0.199	0.224	0.209	0.258	0.198	0.293
	Oracle	0.200	0.032	0.200	0.037	0.198	0.057
80	IVW	0.293	0.108	0.471	0.198	0.664	0.249
	Reg	0.214	0.056	0.253	0.106	0.313	0.160
	Post-reg	0.202	0.068	0.222	0.117	0.260	0.164
	MV-All	0.209	0.188	0.222	0.262	0.235	0.318
	Oracle	0.193	0.038	0.201	0.069	0.217	0.109

Table S5. Mean and standard deviation (SD) of estimates from the various methods when there is sparsity in the covariate effects on the outcome, $\theta = 0.2$, $p = 200$ and $k = 90$.

	$\theta = 0.2$					
	7 Covariates		21 Covariates		35 Covariates	
Method	Mean	SD	Mean	SD	Mean	
			$\gamma_{Wj} = 1/k$			
IVW	0.286	0.108	0.470	0.179	0.666	0.236
Reg	0.192	0.031	0.194	0.046	0.200	0.057
Post-reg	0.172	0.040	0.166	0.054	0.168	0.063
MV-All	0.166	0.053	0.163	0.062	0.167	0.067
Oracle	0.190	0.021	0.185	0.034	0.183	0.045
			$\gamma_{Wj} = 0.5$			
IVW	0.286	0.108	0.470	0.179	0.666	0.236
Reg	0.207	0.035	0.232	0.060	0.268	0.084
Post-reg	0.200	0.043	0.216	0.067	0.238	0.088
MV-All	0.204	0.060	0.218	0.077	0.240	0.094
Oracle	0.193	0.022	0.198	0.040	0.215	0.060

5×10^{-8}, one variant (rs164009) which had a p-value larger than 5×10^{-8}, and less than 5×10^{-7}, was also included on the basis of a known biological role in urate metabolism. The associations between the genetic variants and coronary heart disease as well as the covariates were taken from GWAS data as summarized in Table S6, and accessed using PhenoScanner (Staley *and others*, 2016; Kamat *and others*, 2019).

Note that the analysis by White *and others* (2016) used the 2013 CARDIoGRAMplusC4D dataset, whereas here we use the 2015 dataset. Similarly, White *and others* (2016) used the 2012

Table S6. Sources of associations between the 31 genetic variants and the covariates.

Trait	Consortium	Study	Sample Size
Coronary heart disease	CARDIoGRAMplusC4D	Nikpay *and others* (2015)	184 305
Fasting glucose	MAGIC	Dupuis *and others* (2010)	46 186
BMI	GIANT	Locke *and others* (2015)	339,224
Type 2 diabetes	DIAGRAM	Scott *and others* (2017)	159 208
HDL cholesterol	GLGC	Willer *and others* (2013)	187 167
LDL cholesterol	GLGC	Willer *and others* (2013)	173 082
Triglycerides	GLGC	Willer *and others* (2013)	177 861
Systolic blood pressure	Neale Lab	2017 results	337 199
Diastolic blood pressure	Neale Lab	2017 results	337 199

dataset from DIAGRAM and the 2010 dataset from GIANT, whereas here we use the 2017 and 2015 datasets, respectively. Finally, White *and others* (2016) obtained genetic variant associations with the blood pressure traits from the ICBP consortium, whereas here we use the 2017 results from the analysis of UK Biobank by the Neale Lab (`http://www.nealelab.is/blog/2017/7/19/rapid-gwas-of-thousands-of-phenotypes-for-337000-samples-in-the-uk-biobank`).

S6. Consistency

There are two notions of consistency relating to the Lasso: consistency in estimation, and consistency in model selection (that is, that the procedure shrinks the correct coefficients, and only those, to zero). As discussed in Section 7, it is the latter which is relevant to the consistency of the post-regularization estimator. However, consistency in model selection is stronger than is needed. As long as the Lasso procedure selects at least the truly non-pleiotropic covariates, then the post-regularization estimator will be consistent. Note that this contrasts with the problem considered by Kang *and others* (2016) and Windmeijer *and others* (2019), where consistent estimation relies on selecting no invalid instruments. In that case, the stronger condition of consistency in model selection is required.

For the Lasso to be consistent in model selection, the irrepresentable condition of Zhao and Yu (2006) must be met. This condition places restrictions on the nature of the correlations

between the important predictor variables (that is, the non-zero elements of the true coefficient vector) and the unimportant predictor variables. If the covariates are independent, the Lasso will be consistent in model selection. This will also be the case if the groups of important and unimportant variables are independent of each other, even if they are correlated within group. If the irrepresentable condition is not satisfied, Meinshausen and Yu (2009) have shown that, under weaker conditions, the Lasso will still select the truly non-zero entries of the true coefficient vector as well as some, but not too many, non-zero entries. These conditions will be met if the number of covariates remains fixed as the number of observations increases.

In the model given by (2.1)–(2.3), the covariates are correlated via their association with the common U. Thus, we cannot assume that the method will necessarily be consistent in model selection. However, assuming the two sample setting, we may expect that, as the number of variants gets large, it will still select at least the truly non-pleiotropic covariates. The simulations reported in Section S4.4 demonstrate that the method performs well when correlation among the covariates is increased, even when the pleiotropic and non-pleiotropic covariate groups are correlated. Although there is some bias in Scenario 2 as the number of pleiotropic covariates increase, this bias reduces when the number of instruments is increased to 200 (see Table S5). These results support the assertion that the method is consistent in estimation.

S7. Correlated instruments

In the case of correlated instruments, the matrix Σ_G is not diagonal, and so cannot be replaced by S in (S1.3). Instead, we can estimate Σ_G by $\hat{\Sigma}_G$, which is the $p \times p$ matrix whose inverse has $(i,j)^{\text{th}}$ element $\sigma_i \sigma_j \rho_{ij}$ where $\sigma_i = \text{se}\left(\hat{\beta}_{Yi}\right)$ and $\rho_{ij} = \text{cor}\left(G_i, G_j\right)$. We then use generalized least squares, that is, we fit the model given by (2.4) where $\varepsilon = \begin{bmatrix}\varepsilon_1 & \cdots & \varepsilon_p\end{bmatrix}'$ is normally distributed with mean zero and covariance matrix $\hat{\Sigma}_G$.

In order to estimate the ρ_{ij}'s, we would need access to individual-level data. If this is not

available, the estimator will remain unbiased if we ignore the correlations and use S as the covariance matrix of ε. In fact, it will be unbiased if we use any positive-definite matrix as the covariance matrix of ε. To see this, consider the form of the estimator given in Section S1, with S replaced by some postive-definite matrix Ω.

$$
\begin{aligned}
\hat{\theta} &= \frac{\left\{\hat{\beta}_X - \hat{\beta}_W\left(\hat{\beta}_W'\Omega\hat{\beta}_W\right)^{-1}\left(\hat{\beta}_W'\Omega\hat{\beta}_X\right)\right\}'\Omega\hat{\beta}_Y}{\left\{\hat{\beta}_X - \hat{\beta}_W\left(\hat{\beta}_W'\Omega\hat{\beta}_W\right)^{-1}\left(\hat{\beta}_W'\Omega\hat{\beta}_X\right)\right\}'\Omega\hat{\beta}_X} \\
&= \frac{\left\{\hat{\beta}_X - \hat{\beta}_W\left(\hat{\beta}_W'\Omega\hat{\beta}_W\right)^{-1}\left(\hat{\beta}_W'\Omega\hat{\beta}_X\right)\right\}'\Omega\left(\theta\hat{\beta}_X + \hat{\beta}_W\delta + \varepsilon\right)}{\left\{\hat{\beta}_X - \hat{\beta}_W\left(\hat{\beta}_W'\Omega\hat{\beta}_W\right)^{-1}\left(\hat{\beta}_W'\Omega\hat{\beta}_X\right)\right\}'S\hat{\beta}_X} \\
&= \theta + \frac{\left\{\hat{\beta}_X - \hat{\beta}_W\left(\hat{\beta}_W'\Omega\hat{\beta}_W\right)^{-1}\left(\hat{\beta}_W'\Omega\hat{\beta}_X\right)\right\}'\Omega\varepsilon}{\left\{\hat{\beta}_X - \hat{\beta}_W\left(\hat{\beta}_W'\Omega\hat{\beta}_W\right)^{-1}\left(\hat{\beta}_W'\Omega\hat{\beta}_X\right)\right\}'\Omega\hat{\beta}_X},
\end{aligned}
$$

and it follows that $E\left(\hat{\theta} - \theta\right) = 0$. The computed standard error of $\hat{\theta}$, however, will not be correct if the instruments are correlated but the correlation is ignored. Note that the above is a straightforward extension of the work of Burgess *and others* (2016) on summarized data methods in Mendelian randomization in the single variable case.

References

Burgess, S., Dudbridge, F. and Thompson, S. G. (2016). Combining information on multiple instrumental variables in Mendelian randomization: comparison of allele score and summarized data methods. *Statistics in Medicine* **35**(11), 1880–1906.

Burgess, S. and Thompson, S. G. (2017). Interpreting findings from Mendelian randomization using the MR-Egger method. *European Journal of Epidemiology* **32**(5), 377–389.

Dupuis, J., Langenberg, C., Prokopenko, I. *and others*. (2010). New genetic loci implicated in fasting glucose homeostasis and their impact on type 2 diabetes risk. *Nature Genetics* **42**(2), 105.

Hastie, T., Tibshirani, R. and Friedman, J. (2009). *The Elements of Statistical Learning: Data Mining, Inference, and Prediction*. New York: Springer.

Jiang, L., Oualkacha, K., Didelez, V. *and others*. (2019). Constrained instruments and their application to Mendelian randomization with pleiotropy. *Genetic Epidemiology* **43**(4), 373–401.

Kamat, M. A, Blackshaw, J. A, Young, R. *and others*. (2019). PhenoScanner V2: an expanded tool for searching human genotype–phenotype associations. *Bioinformatics*.

Kang, H., Zhang, A., Cai, T. T. and Small, D. S. (2016). Instrumental variables estimation with some invalid instruments and its application to Mendelian randomization. *Journal of the American Statistical Association* **111**(513), 132–144.

Locke, A. E., Kahali, B., Berndt, S. I. *and others*. (2015). Genetic studies of body mass index yield new insights for obesity biology. *Nature* **518**(7538), 197.

Meinshausen, Nicolai and Yu, Bin. (2009). Lasso-type recovery of sparse representations for high-dimensional data. *The Annals of Statistics* **37**(1), 246–270.

Nikpay, M., Goel, A., Won, H. H. *and others*. (2015). A comprehensive 1000 genomes–based genome-wide association meta-analysis of coronary artery disease. *Nature Genetics* **47**(10), 1121.

Scott, R. A., Scott, L. J., Mägi, R. *and others*. (2017). An expanded genome-wide association study of type 2 diabetes in Europeans. *Diabetes* **66**(11), 2888–2902.

Staley, J. R., Blackshaw, J., Kamat, M. A. *and others*. (2016). PhenoScanner: a database of human genotype–phenotype associations. *Bioinformatics* **32**(20), 3207–3209.

Thompson, S. G. and Sharp, S. J. (1999). Explaining heterogeneity in meta-analysis: a comparison of methods. *Statistics in Medicine* **18**(20), 2693–2708.

White, J., Sofat, R., Hemani, G. *and others*. (2016). Plasma urate concentration and risk of coronary heart disease: a Mendelian randomisation analysis. *The Lancet Diabetes & Endocrinology* **4**(4), 327 – 336.

Willer, C. J., Schmidt, E. M., Sengupta, S. *and others*. (2013). Discovery and refinement of loci associated with lipid levels. *Nature Genetics* **45**(11), 1274.

Windmeijer, F., Farbmacher, H., Davies, N. and Davey Smith, G. (2019). On the use of the Lasso for instrumental variables estimation with some invalid instruments. *Journal of the American Statistical Association* **114**(527), 1339–1350.

Zhao, Peng and Yu, Bin. (2006). On model selection consistency of Lasso. *Journal of Machine Learning Research* **7**(Nov), 2541–2563.
